# Sex-Specific Coronary Artery Calcium Score Threshold Predictive of Obstructive Coronary Artery Disease

**DOI:** 10.1016/j.jscai.2025.104048

**Published:** 2025-12-18

**Authors:** Denizhan Ozdemir, Devarshi Vasa, Serdar Farhan, Manish Vinayak, James Johnson, Vishal Dhulipala, Amir Hamdan, Gina LaRocca, Annapoorna S. Kini, Deepak L. Bhatt, Samin K. Sharma

**Affiliations:** The Zena and Michael A. Wiener Cardiovascular Institute, Mount Sinai Fuster Heart Hospital, Icahn School of Medicine at Mount Sinai, New York, New York

**Keywords:** coronary artery calcium score, obstructive coronary artery disease, sex-specific coronary artery calcium score threshold

## Abstract

**Background:**

Coronary computed tomography angiography and coronary artery calcium score (CACS) are rapidly being adopted for the noninvasive assessment of coronary artery disease (CAD). One of the primary limitations of calcium imaging is the inability to assess obstructive lesions in the presence of a high calcium burden. Currently, there is no guidance on what level of coronary artery calcium indicates a high risk of obstructive CAD. We aimed to determine sex-specific CACS thresholds suggestive of obstructive CAD by comparing coronary angiography with adjunctive tools to available CACS information.

**Methods:**

From August 2018 to August 2023, we retrospectively analyzed 1799 consecutive patients' clinical characteristics, angiographic information, and available intracoronary physiological/anatomical testing at a single institution. We evaluated the sex-specific distribution of CACS and its specificity for identifying obstructive CAD at a threshold of 90%.

**Results:**

Baseline characteristics were similar between the obstructive (n = 1223) and nonobstructive (n = 576) CAD groups. A CACS of ≥1000 in women (HR, 2.81; 95% CI, 1.77-4.47; *P* < .001) and ≥1400 in men (HR, 3.34; 95% CI, 2.23-5.02; *P* < .001) predicted obstructive CAD at 90% specificity.

**Conclusions:**

A CACS of ≥1000 in women and ≥1400 in men identifies obstructive CAD at 90% specificity. These thresholds should be prospectively validated and may potentially be used to guide the selection of patients who would benefit from intensification of medical therapy or invasive evaluation in the appropriate clinical context.

## Introduction

Coronary artery disease (CAD) remains a leading cause of morbidity and mortality globally, with an estimated 1 in 4 deaths being due to CAD.^1^^,^^2^ The CAD often presents as subclinical atherosclerosis before manifesting as symptomatic disease, making early detection of CAD crucial for implementing preventive measures and reducing cardiovascular morbidity and mortality.^1^ Therefore, the assessment of subclinical atherosclerotic disease has become an essential goal in preventive cardiology.^3^ Coronary artery calcification is a hallmark of atherosclerosis, posing a worse prognosis for cardiovascular events.^3^ With advances in imaging, coronary artery calcification has become a direct biomarker for risk-stratifying patients for short- and long-term adverse cardiac events.^3^^,^^4^ Furthermore, clinical guidelines in the United States and Europe consider the coronary artery calcium score (CACS) to be a useful way of improving cardiovascular risk assessment in asymptomatic patients for risk stratification and serving as a guide for initiating or deferring preventive therapies.^5^^,^^6^ Currently, CACS can refine the risk assessment in patients with intermediate risk (5%-20%) as predicted by the pooled cohort equations.^6^ Visually estimated CACS, a complementary approach, has become a routine part of noninvasive stress test modalities, including single photon emission computed tomography myocardial perfusion imaging (SPECT MPI), positron emission tomography (PET)/CT, and coronary computed tomography angiography (CTA), enhancing diagnostic information.^7^^,^^8^ Importantly, prior research has demonstrated sex-based differences in calcium deposition and plaque morphology, with women more likely to have lower CACS compared to men. This supports the need for sex-specific thresholds to optimize diagnostic accuracy and avoid underestimating CAD burden in women.^9^^,^^10^

Beyond its role in CAD risk stratification, similar principles have been applied to aortic valve calcium scoring, with the measurement of cumulative calcium burden being well-established in aortic stenosis.^11^ An aortic valve calcium score of ≥2000 in men and ≥1300 in women indicates a high likelihood of severe aortic stenosis, especially when echocardiographic parameters are inconclusive, such as in low-flow, low-gradient cases, or when imaging is suboptimal.^11^^,^^12^ This association between calcium burden and disease severity supports the premise of our study.

A systematic review reported the specificity of different noninvasive physiological modalities, including SPECT MPI, PET/CT, stress echocardiography, and stress cardiac magnetic resonance imaging, as follows: exercise stress electrocardiogram specificity ranged from 64% to 85% for women and 74% to 83% for men; stress echocardiography ranged from 79% to 92% for women and 84% to 93% for men; SPECT MPI ranged from 58% to 91% for women and 62% to 89% for men; and cardiac MRI ranged from 81% to 88% for women and 74% to 94% for men.^13^ Although coronary CTA has excellent sensitivity for ruling out obstructive CAD, high CACS impairs its ability to diagnose obstructive CAD due to blooming artifacts.^14^ Therefore, it is currently unclear at what level of CACS there is a high likelihood of obstructive CAD compared with coronary angiography. Given the established specificity levels of various noninvasive modalities and their limitations,^13^ our study aimed to identify obstructive CAD using CACS at 90% specificity—a threshold higher than that of commonly used modalities—following invasive evaluation with coronary angiography and adjunctive tools as needed.

## Materials and methods

### Patients

Between August 2018 and 2023, our study analyzed 1799 patients who were referred to the Mount Sinai Hospital Cardiac Catheterization Laboratory with available CACS information as part of their prior evaluation. Patients with symptoms concerning angina who had undergone previous evaluation with available CACS were included in this retrospective study. We estimate that the majority of our patients were at low to intermediate risk based on their pretest probability. The study was approved by the local ethics committee and met IRB standards (IRB identifier STUDY-23-01615 Calcium Score Threshold for Obstructive CAD). Given the retrospective nature of our research, a waiver of consent was obtained in compliance with institutional standards. Our study excluded patients with human immunodeficiency virus infection, as well as those enrolled in investigational drug or device trials, in accordance with institutional IRB protocols.

### CACS and invasive coronary angiography

Our center and catheterization laboratory serve as a referral hub in a large metropolitan area. An internal software system allows us to capture the total CACS as part of the pertinent clinical information. However, vessel-specific CACS is not routinely available due to variations in reporting systems from a large number of referring sources.

Invasive angiography images are evaluated in a standardized manner, with significant lesions defined as ≥50% stenosis in the left main and ≥70% stenosis in other major coronary arteries. In cases of intermediate or equivocal lesions, adjunctive imaging and physiological testing are utilized to determine the significance of the lesions, which are then reclassified as nonobstructive or obstructive in accordance with the 2021 ACC/AHA/SCAI revascularization guidelines. Clinical, imaging, and angiographic characteristics were extracted from electronic medical records. Patients were categorized into obstructive (n = 1223) and nonobstructive (n = 576) CAD based on angiographic criteria. CACS values were extracted from computed tomography imaging reports. Data accuracy was verified by 2 independent investigators to ensure reliability.

### Study end points

Our aim was to define a high-specificity threshold of 90%, based on the current specificity levels of other noninvasive modalities, by using coronary angiography with adjunctive tools in a large dataset. To the best of our knowledge, this study presents the first and largest dataset with available CACS and coronary angiogram correlations. We classified our cohort into nonobstructive and obstructive CAD based on the coronary angiogram and accepted guidelines.

We then performed a specificity analysis for different levels of CACS, considering all age groups, as well as an exploratory analysis of patients under the age of 65 years and those aged 65 years or older.

### Quantitative coronary angiography and adjudication

Coronary angiograms were reviewed by 2 independent investigators. A random selection of 50 consecutive angiographic images was taken from data collected every August between August 2018 and August 2022, for a total of 250 patients. This was performed to ensure that quantitative coronary angiography (QCA) and clinical assessments were within acceptable limits of concordance. For each patient, all 3 major coronary arteries (left anterior descending artery, left circumflex artery, right coronary artery, as well as the ramus intermedius, if present) were assessed using QAngio software (Medis Medical), with stenosis considered obstructive if it was measured at ≥50%. Clinical assessments of obstructive versus nonobstructive classification were made based on current guidelines. A kappa test was applied to assess the agreement between the clinical classification (obstructive, nonobstructive) and the QCA analysis.

### Statistical analysis

Continuous variables are expressed as the number of observations, mean ± SD, or median (interquartile range). *P* values for continuous variables were analyzed using either *t* test or the Mann-Whitney U test, as appropriate. Categorical and dichotomous variables are summarized as counts and percentages with 95% CIs and were compared using the χ^2^ test. Fisher exact test was used for categorical values with counts less than 5. Statistical significance was defined as a *P* value < .05. Sex-specific specificity testing was applied at a 90% threshold for obstructive CAD, a level comparable to or greater than that of current noninvasive stress testing modalities. All data were analyzed using Stata software.

## Results

### Baseline characteristics

A total of 1799 patients who underwent both CACS assessment and coronary angiography between 2018 and 2022 were included in the study. Baseline clinical characteristics are shown in Table 1. Compared with patients with nonobstructive CAD, there was a higher proportion of hyperlipidemia, diabetes, and a family history of CAD in patients with obstructive CAD (Table 1). In contrast, there was a lower proportion of women and atrial fibrillation among those with obstructive CAD compared with those with nonobstructive CAD (Table 1). Otherwise, the groups were similar with respect to age, race/ethnicity, body surface area, and comorbidities (obesity, hypertension, current or former smoking, history of heart failure, chronic kidney disease, end-stage renal disease, and asthma) (Table 1). Left ventricular ejection fraction was similar between the groups.

**Table 1:** Baseline characteristic of nonobstructive and obstructive CAD groups.

	Nonobstructive CAD (n = 576)	Obstructive CAD (n = 1223)	*P* value
Age, y	66.8 ± 10	66.2 ± 10	.87
Female sex	46% (265)	30% (365)	.01
Race/ethnicity			
White	59% (343)	63% (777)	–
African American	11% (65)	7% (85)	–
Hispanic	19% (108)	15% (184)	–
South Asian	8% (47)	12% (150)	–
East Asian	2% (13)	2% (25)	–
BSA, m^2^	1.9 ± 0.4	1.9 ± 0.3	.72
Obese	19% (109)	21% (265)	.18
Hypertension	79% (457)	80% (986)	.53
Hyperlipidemia	77% (447)	85% (1043)	.001
Diabetes	29% (170)	36% (441)	.006
Smoker - current	8% (47)	9% (106)	.72
Smoker - former	24% (142)	27% (327)	.35
Family history of CAD	28% (163)	34% (411)	.02
History of heart failure	11% (65)	8% (98)	.2
Atrial fibrillation	12% (71)	6% (74)	.01
Chronic kidney disease	12% (71)	14% (171)	.33
ESRD/dialysis	<1% (5)	<1% (6)	.91
Asthma	12% (70)	10% (123)	.18
LVEF, %	61 ± 8	60 ± 8	.86

BSA, body surface area; CAD, coronary artery disease; ESRD, end-stage renal disease; LVEF, left ventricular ejection fraction.

### Specificity and sensitivity analyses

**Figure 1:**
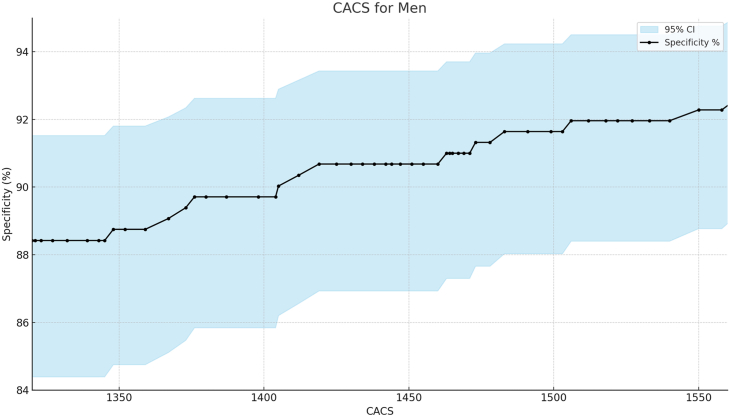
**Specificity analysis for obstructive coronary artery disease and coronary artery calcium score (CACS) in men.** Specificity values with 95% CI for varying CACS thresholds. A threshold of ≥1400 was associated with 90% specificity.

**Figure 2:**
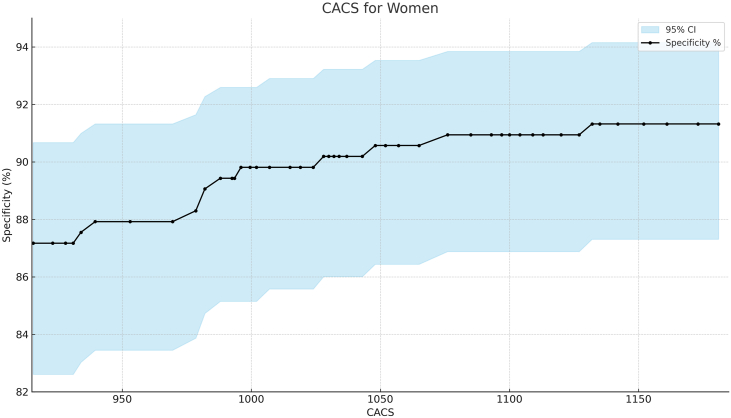
**Specificity analysis for obstructive coronary artery disease and coronary artery calcium score (CACS) in women.** Graphical representation of specificity at increasing CACS thresholds. A CACS ≥1000 identified obstructive CAD at 90% specificity.

We performed a specificity analysis to determine the CACS threshold for identifying obstructive CAD, establishing 90% as the cutoff based on the specificity levels of other noninvasive modalities. Across all age groups, men (n = 1169) and women (n = 630) had CACS thresholds of ≥1400 (Figure 1) and ≥1000 (Figure 2), respectively, which identified obstructive CAD at 90% specificity. Varying levels of specificity, along with 95% CIs for both sexes, are illustrated in Figures 1 and 2. In addition, we performed sensitivity, positive predictive value, and negative predictive value analyses for CACS thresholds of ≥1400 for men and ≥1000 for women. For men, we found a sensitivity of 27%, a positive predictive value of 88%, and a negative predictive value of 30%. For women, the sensitivity was 26%, the positive predictive value was 78%, and the negative predictive value was 47%.

### Distribution of CACS

**Figure 3:**
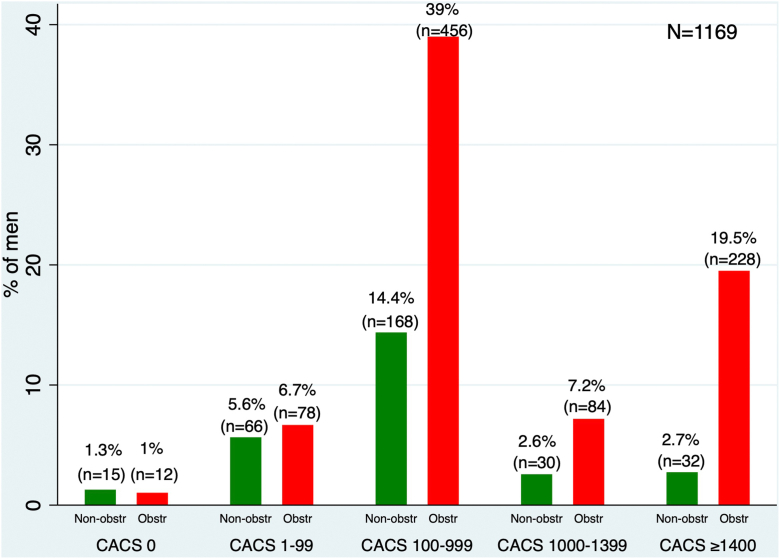
**Distribution of coronary artery calcium score (CACS) in men with obstructive (Obstr) and nonobstructive (Non-obstr) coronary artery disease.** CACS stratified by group and annotated with the 90% specificity threshold (≥1400) to highlight group differences.

**Figure 4:**
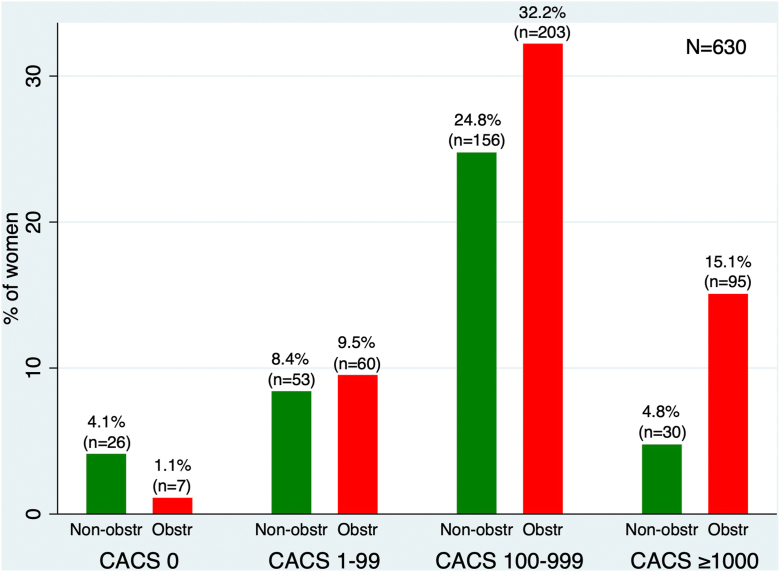
**Distribution of coronary artery calcium score (CACS) in women with obstructive (Obstr) and nonobstructive (Non-obstr) coronary artery disease** (**CAD).** CACS stratified by group with the 90% specificity threshold (≥1000) indicated to compare distributions.

Overall, 1% of men and 1.1% of women had a CACS of 0 but were found to have obstructive CAD after angiographic assessment. The proportion of obstructive CAD with a CACS of 0 was similar to that reported in a large international multicenter study (CONFIRM: Coronary CT Angiography Evaluation for Clinical Outcomes: an International Multicenter Registry), which found a similar proportion of ≥70% coronary stenosis in 67 patients out of 4738, corresponding to 1.4%.^15^ We used commonly accepted CACS thresholds for risk stratification and patient management, specifically CACS 0, 1 to 99, and 100 to 999.^4^ Based on our specificity thresholds for obstructive CAD, we illustrated the respective cutoffs: a CACS of ≥1400 for men (Figure 3) and ≥1000 for women (Figure 4) in the distribution of CACS.

### Predictors of obstructive CAD

Univariate predictors of obstructive CAD for men are listed in Table 2 and include clinical risk factors, race/ethnicity, and CACS ≥1400. In multivariate analysis, CACS ≥1400 was a strong predictor of obstructive CAD (HR, 3.34; 95% CI, 2.23-5.02; *P* < .001). Additionally, for men, hyperlipidemia (HR, 1.50; 95% CI, 1.06-2.10; *P* = 0.020), obesity (HR, 1.46; 95% CI, 1.02-2.10; *P* = .04), and atrial fibrillation (HR, 0.34; 95% CI, 0.22-0.52; *P* = .001) remained statistically significant.

**Table 2:** Univariable and multivariable hazard ratio estimates for obstructive CAD among men.

Variable	Univariable analysis		Multivariable analysis	
	HR (95% CI)	*P* value	HR (95% CI)	*P* value
Age at procedure	1.00 (0.99-1.01)	.694	–	–
BSA	0.85 (0.60-1.21)	.379	–	–
Hypertension	1.19 (0.88-1.62)	.251	–	–
Diabetes	1.14 (0.86-1.50)	.366	–	–
Hyperlipidemia	1.62 (1.17-2.26)	.003^a^	1.50 (1.06-2.10)	.020^a^
Smoker (current)	0.93 (0.61-1.42)	.742	–	–
Smoker (former)	1.22 (0.90-1.64)	.197	–	–
Family history	1.30 (1.05-1.86)	.022^a^	1.27 (0.94-1.70)	.118
Obese	1.59 (1.11-2.26)	.010^a^	1.46 (1.02-2.10)	.04^a^
Atrial fibrillation	0.35 (0.23-0.53)	.001^a^	0.34 (0.22-0.52)	.001^a^
Asthma	0.88 (0.55-1.40)	.584		
LVEF	1.00 (0.98-1.02)	.98	–	–
CKD	1.29 (0.83-2.00)	.259	–	–
CACS ≥1400	3.16 (2.13-4.70)	<.001^a^	3.34 (2.23-5.02)	<.001^a^
White	1.03 (0.78-1.36)	.812	–	–
African American	0.84 (0.45-1.56)	.581	–	–
East Asian	0.682 (0.31-1.48)	.335	–	–
South Asian	1.27 (0.84-1.91)	.261	–	–
Other race	0.88 (0.61-1.28)	.525	–	–

BSA, body surface area; CACS, coronary artery calcium score; CAD, coronary artery disease; CKD, chronic kidney disease; LVEF, left ventricular ejection fraction.

a This variable was found to be statistically significant in the univariate and/or multivariate analyses.

Univariate predictors of obstructive CAD for women are also listed in Table 3 and include clinical risk factors, race/ethnicity, and CACS ≥1000. In multivariate analysis, CACS ≥1000 was a strong predictor of obstructive CAD (HR, 2.81; 95% CI, 1.77-4.47; *P* < .001). Additionally, diabetes (HR, 1.63; 95% CI, 1.15-2.32; *P* = 0.006) and hyperlipidemia (HR, 1.55; 95% CI, 1.02-2.37; *P* = .04) remained statistically significant in women.

**Table 3:** Univariable and multivariable hazard ratio estimates for obstructive CAD among women.

Variable	Univariable analysis		Multivariable analysis	
	HR (95% CI)	*P* value	HR (95% CI)	*P* value
Age at procedure	1.00 (0.99-1.02)	.466	–	–
BSA	0.56 (0.28-1.14)	.110	–	–
Hypertension	1.16 (0.74-1.80)	.521	–	–
Diabetes	1.89 (1.34-2.64)	.001^a^	1.63 (1.15-2.32)	.006^a^
Hyperlipidemia	1.76 (1.17-2.64)	.006^a^	1.55 (1.02-2.37)	.040^a^
Smoker (current)	1.01 (0.48-2.10)	.982	–	–
Smoker (former)	0.90 (0.62-1.31)	.595	–	–
Family history	1.10 (0.79-1.56)	.562	–	–
Obese	0.90 (0.62-1.32)	.595	–	–
Atrial fibrillation	0.68 (0.37-1.24)	.207	–	–
LVEF	0.99 (0.97-1.02)	.575	–	–
CKD	1.32 (0.87-1.99)	.192	–	–
CACS ≥1000	2.95 (1.87-4.65)	<.001^a^	2.81 (1.77-4.47)	<.001^a^
White	1.18 (0.86-1.62)	.311	–	–
African American	0.69 (0.45-1.05)	.083	–	–
East Asian	1.46 (0.36-5.91)	.592	–	–
South Asian	2.09 (1.11-3.94)	.022^a^	1.86 (0.96-3.59)	.065
Other race	0.75 (0.51-1.11)	.156	–	–

BSA, body surface area; CACS, coronary artery calcium score; CAD, coronary artery disease; CKD, chronic kidney disease; LVEF, left ventricular ejection fraction.

a This variable was found to be statistically significant in the univariate and multivariate analyses.

### Kappa test for adjudication

We analyzed a total of 763 coronary arteries for adjudication from 250 patients (including the 3 major epicardial coronary arteries and the ramus intermedius, if present). Fifty consecutive patients were selected through random sampling each August, from August 1, 2018, to August 2022. The accuracy of agreement between QCA and clinical assessment in our study was 86%, with a kappa score of 0.65, indicating substantial agreement (Supplemental Figure S1).

### Age subgroup analysis for patients under and above 65 years of age

We conducted a separate analysis based on age groups, dividing the cohort into those younger than 65 years and those aged 65 years and older, to determine if CACS thresholds vary by age. The study population was divided into 4 groups based on sex and age, and their respective CACS thresholds were analyzed. Among men under the age of 65 years (n = 565), 487 individuals (86.2%) had a CACS <1400, whereas 78 individuals (13.8%) had a CACS ≥1400. For men aged 65 years and older (n = 604), 422 individuals (69.9%) had a CACS <1400, whereas 182 individuals (30.1%) had a CACS ≥1400.

For women under the age of 65 years (n = 209), 176 individuals (84.2%) had a CACS <1000, and 33 individuals (15.8%) had a CACS ≥1000. Among women aged 65 years and older (n = 421), 332 individuals (78.9%) had a CACS <1000, whereas 89 individuals (21.1%) had a CACS ≥1000.

For men younger than 65 years, the odds ratio for CACS ≥1400 in predicting obstructive CAD was 2.77 (95% CI, 1.39-5.53), whereas for men aged 65 years or older, the odds ratio was 3.53 (95% CI, 2.17-5.77), with a *P* value for age subgroup interaction of 0.58 (Supplemental Figure S2). For women younger than 65 years, the odds ratio for CACS ≥1000 in predicting obstructive CAD was 3.24 (95% CI, 1.34-7.86), and for women aged 65 years or older, the odds ratio was 2.84 (95% CI, 1.66-4.84), with a *P* value for age subgroup interaction of 0.80 (Supplemental Figure S3).

## Discussion

In this large, single-center study of 1799 patients undergoing both CACS and invasive coronary angiography, we found that a CACS ≥1400 in men and ≥1000 in women identified obstructive CAD with 90% specificity. These sex-specific thresholds may aid in clinical decision-making, particularly for selecting patients who may benefit from further testing or intensification of therapy.

The aortic valve calcium score provides diagnostic information in cases of equivocal data or as an independent criterion for diagnosing aortic stenosis (≥2000 for men and ≥1300 for women), given that aortic valve calcification is a mechanistic and pathophysiologic process of aortic stenosis.^11^^,^^12^ Our study is based on the premise that coronary artery calcification is the hallmark of atherosclerosis with a measurable threshold effect. Current consensus statements recommend cholesterol-lowering therapy for any CACS greater than 0, and antiplatelet therapy for a CACS greater than 99.^4^^,^^16^ Previous studies have shown a higher likelihood of abnormal myocardial perfusion imaging if CACS is ≥400.^17^

Yuoness et al^18^ previously identified 26 patients with CACS >1000 and normal SPECT MPI results, of whom 15 (58%) had severe disease, defined as ≥70% stenosis. Because of the small sample size of the study, no sex-specific CACS thresholds were established. Pedersen et al^19^ reported a low prevalence of obstructive CAD (1.8%) when CACS = 0 in patients at low to intermediate risk; however, their study did not report the specific values identified in our research. Nicoll et al^20^ reported that coronary stenosis, defined as ≥50% on coronary CTA in the study, is most accurately predicted by increasing CACS, particularly up to 400, when risk factors are considered. However, this study did not establish sex-specific thresholds or utilize invasive coronary angiography. Chen et al^21^ investigated a range of CACS in 208 patients to identify thresholds compared with invasive coronary angiography, using invasive stenosis thresholds of 50% and 70%. They identified 10 patients with CACS ≥1000 and 7 patients with CACS ≥1300. Notably, all 7 patients with CACS ≥1300 demonstrated 50% stenosis at 100% specificity. This study did not establish sex-specific thresholds due to the small sample size.^21^

Currently utilized noninvasive physiologic test modalities (SPECT MPI, PET/CT, stress echocardiography, stress cardiac magnetic resonance imaging) provide varying levels of specificity as previously discussed.^13^ Severe coronary artery calcification is the Achilles’ heel of coronary CTA, as it causes overestimation of coronary stenosis.^22^ Given that CACS information is obtained during CTA, our study investigates the utility of a high CACS for risk stratification in diagnosing obstructive CAD, aiming to establish a specificity threshold comparable to or greater than that of current noninvasive modalities.

We hypothesized that an already available CACS threshold can identify patients at increased risk of obstructive CAD, thereby optimizing patient risk stratification, elective triage for invasive coronary procedures, and potentially streamlining the diagnostic pathway for those requiring revascularization in the appropriate clinical context. Through a comprehensive analysis of CACS in a large cohort of 1799 patients, we demonstrated that a sex-specific CACS threshold of ≥1400 in men and ≥1000 in women suggests 90% specificity in our cohort. In addition, our analysis suggests that there is no age subgroup interaction between patients younger or older than 65 years of age for these thresholds in the respective sexes. Our study builds upon previously reported findings in smaller cohorts that lacked sex-specific cutoffs or direct comparisons to invasive coronary angiography. We identified sex-specific cutoffs in a large patient cohort to evaluate CACS thresholds at 90% specificity, compared with the gold standard of invasive coronary angiography, using adjunctive tools. These findings may assist clinicians in identifying patients who could benefit from more intensive secondary prevention strategies or further diagnostic evaluation, particularly in cases where symptoms or noninvasive testing are inconclusive. Although current guidelines already recommend preventive therapy for patients with CACS >100, our results suggest that very high calcium burdens of ≥1000 in women and ≥1400 in men may indicate a higher likelihood of obstructive CAD and prompt consideration of invasive coronary angiography in the appropriate clinical context. However, these thresholds must be integrated into a comprehensive clinical framework that includes symptom assessment, risk factor assessment, and other diagnostic modalities. The study also underscores the need for prospective validation to determine whether such thresholds can effectively guide therapeutic escalation with agents like GLP-1 receptor agonists, PCSK9 inhibitors, or coronary revascularization.

In our study, the accuracy between quantitative coronary angiography and clinical assessment was 86%, with a kappa score of 0.65, indicating substantial agreement and strong internal validity. This result is comparable to an analysis from the PROMISE trial, which found a disagreement rate of 19.1% and a kappa score of 0.63.^23^

Visually estimated CACS has already been implemented from CT attenuation for PET/CT and assists clinicians in providing further risk estimation. As this information becomes more readily accessible from noncoronary CT studies (such as lung nodule surveillance or CTA for pulmonary embolism), it may serve as an additional data point for clinicians managing and optimizing secondary risk reduction therapies.^24^

The strengths of our study include a large cohort of patients, a CACS distribution comparable to contemporary CT studies,^15^ the direct comparison of CACS with invasive angiography, and a substantial agreement rate between quantitative coronary angiography and clinical assessment. Our study holds promise as a readily available noninvasive tool for identifying individuals who may benefit from further evaluation, particularly those being considered for the escalation of optimal medical therapy or further downstream testing.

### Study limitations

Our study has several limitations. First, our database is limited to a single large tertiary referral center and was performed retrospectively, which may affect generalizability, and these results should be prospectively validated in diverse clinical settings. Second, our study does not provide a direct head-to-head comparison between other noninvasive stress test modalities and CACS; hence, it is unclear whether a CACS threshold of ≥1400 in men and ≥1000 in women would provide superior accuracy compared with physiological stress testing in diagnosing obstructive CAD. Third, due to the variety of referral sources to our center, our database lacks vessel-specific CACS, preventing us from deducing vessel-specific thresholds for the left anterior descending artery, right coronary artery, left circumflex artery, or ramus intermedius, when present. Fourth, it is well known that CACS increases over time, particularly with the use of statins. Due to the retrospective nature of our study and the lack of precise timing between CACS and coronary angiography, we cannot rule out the possibility of coronary stenosis progression over time, which may affect the specificity and accuracy of CACS. Fifth, although the Agatston score is widely recognized for its prognostic value, it is limited by its reliance on peak Hounsfield units. Additionally, due to the retrospective design of our study and the use of multiple data sources, we were unable to incorporate mass and volume scores, which could provide a more comprehensive assessment of calcium content. Sixth, our study included only patients undergoing coronary angiography. Therefore, it is plausible that patients identified with nonobstructive findings on CTA may not proceed to angiography, which could affect the accuracy of these thresholds. Seventh, our study did not assess the incremental diagnostic value of CACS over CTA findings, or the contribution of coronary CTA and CACS in diagnosing obstructive CAD, due to variability in reporting. Additionally, although the specificity of CACS ≥1000 in women was 90%, the positive predictive value was modest at 78%. This may reflect the lower calcium burden typically observed in women despite significant disease and highlights the importance of multimodal evaluation. It is also important to note the low sensitivity of the identified CACS thresholds. Although we found that a CACS ≥1400 in men and ≥1000 in women identifies obstructive CAD at 90% specificity, the corresponding sensitivities were only 27% and 26%, respectively. This means that the majority of patients with obstructive CAD fall below these thresholds, limiting their use as screening tools. These thresholds may be more useful for confirming high risk in patients already under consideration for further testing or treatment (Central Illustration). Lastly, most of the patients with obstructive CAD fell under our recommended sex-specific CACS thresholds. Men with CACS ≥1400 represented 27% of all men with obstructive CAD (228 out of 858 patients). Similarly, women with CACS ≥1000 represented 26% of all women with obstructive CAD (95 out of 365 patients). These thresholds should be interpreted with caution, as these specific patient subgroups likely represent those who may benefit from the up-titration of medical therapy. In the absence of head-to-head data, these thresholds should not override medical decision-making based on current guidelines regarding intervention or surgical revascularization.

**Central Illustration:**
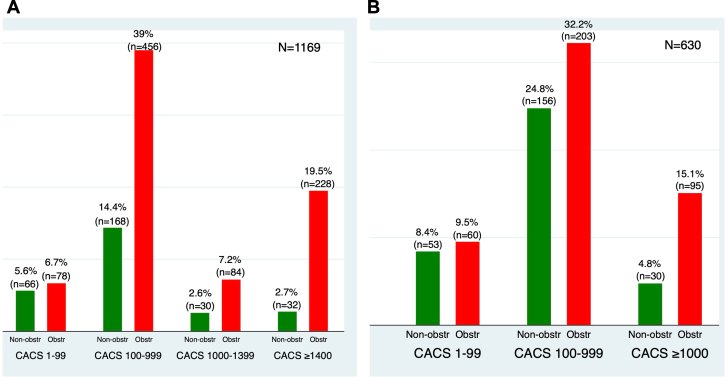
Distribution of coronary artery calcium score (CACS) in men and women with obstructive (Obstr) and nonobstructive (Non-obstr) coronary artery disease (CAD).

## Conclusion

The present study demonstrates the incremental value of high CACS thresholds (≥1400 in men and ≥1000 in women) for establishing sex-specific thresholds to diagnose obstructive CAD. This helps mitigate the inherent limitations of coronary CT imaging due to high calcium burden and may guide clinicians in optimizing secondary prevention therapies and/or assist in selecting downstream testing in correlation with the gold standard of invasive coronary angiography and adjunctive intracoronary physiological testing or imaging. These findings should be prospectively validated.

## Declaration of competing interest

Deepak L. Bhatt discloses the following relationships: Advisory board – AngioWave, Bayer, Boehringer Ingelheim, CellProthera, Cereno Scientific, Elsevier Practice Update Cardiology, High Enroll, Janssen, Level Ex, McKinsey, Medscape Cardiology, Merck, MyoKardia, NirvaMed, Novo Nordisk, PhaseBio, PLx Pharma, and Stasys; board of directors – American Heart Association, New York City, AngioWave (stock options), Bristol-Myers Squibb (stock), DRS.LINQ (stock options), and High Enroll (stock); consultant – Broadview Ventures, GlaxoSmithKline, Hims, SFJ, and Youngene; data monitoring committees – Acesion Pharma, Assistance Publique-Hôpitaux de Paris, Baim Institute for Clinical Research (formerly Harvard Clinical Research Institute, for the PORTICO trial, funded by St. Jude Medical, now Abbott), Boston Scientific (Chair, PEITHO trial), Cleveland Clinic, Contego Medical (Chair, PERFORMANCE 2), Duke Clinical Research Institute, Mayo Clinic, Mount Sinai School of Medicine (for the ENVISAGE trial, funded by Daiichi Sankyo; for the ABILITY-DM trial, funded by Concept Medical; for ALLAY-HF, funded by Alleviant Medical), Novartis, Population Health Research Institute; Rutgers University (for the NIH-funded MINT trial); honoraria – American College of Cardiology (Senior Associate Editor, Clinical Trials and News, ACC.org; Chair, ACC Accreditation Oversight Committee), Arnold and Porter law firm (work related to Sanofi/Bristol-Myers Squibb clopidogrel litigation), Baim Institute for Clinical Research (formerly Harvard Clinical Research Institute; RE-DUAL PCI clinical trial steering committee funded by Boehringer Ingelheim; AEGIS-II executive committee funded by CSL Behring), Belvoir Publications (Editor in Chief, Harvard Heart Letter), Canadian Medical and Surgical Knowledge Translation Research Group (clinical trial steering committees), CSL Behring (AHA lecture), Cowen and Company, Duke Clinical Research Institute (clinical trial steering committees, including for the PRONOUNCE trial, funded by Ferring Pharmaceuticals), HMP Global (Editor in Chief, *Journal of Invasive Cardiology*), *Journal of the American College of Cardiology* (Guest Editor; Associate Editor), K2P (Co-Chair, interdisciplinary curriculum), Level Ex, Medtelligence/ReachMD (CME steering committees), MJH Life Sciences, Oakstone CME (Course Director, Comprehensive Review of Interventional Cardiology), Piper Sandler, Population Health Research Institute (for the COMPASS operations committee, publications committee, steering committee, and USA national co-leader, funded by Bayer), WebMD (CME steering committees), Wiley (steering committee); other: *Clinical Cardiology* (Deputy Editor); patent: Sotagliflozin (named on a patent for sotagliflozin assigned to Brigham and Women's Hospital who assigned to Lexicon; neither the author nor Brigham and Women's Hospital receive any income from this patent); research funding – Abbott, Acesion Pharma, Afimmune, Aker Biomarine, Alnylam, Amarin, Amgen, AstraZeneca, Bayer, Beren, Boehringer Ingelheim, Boston Scientific, Bristol-Myers Squibb, Cardax, CellProthera, Cereno Scientific, Chiesi, CinCor, Cleerly, CSL Behring, Eisai, Ethicon, Faraday Pharmaceuticals, Ferring Pharmaceuticals, Forest Laboratories, Fractyl, Garmin, HLS Therapeutics, Idorsia, Ironwood, Ischemix, Janssen, Javelin, Lexicon, Lilly, Medtronic, Merck, Moderna, MyoKardia, NirvaMed, Novartis, Novo Nordisk, Otsuka, Owkin, Pfizer, PhaseBio, PLx Pharma, Recardio, Regeneron, Reid Hoffman Foundation, Roche, Sanofi, Stasys, Synaptic, The Medicines Company, Youngene, and 89Bio; royalties: Elsevier (Editor, *Braunwald’s Heart Disease*); site co-investigator – Abbott, Biotronik, Boston Scientific, CSI, Endotronix, St. Jude Medical (now Abbott), Philips, SpectraWAVE, Svelte, and Vascular Solutions; trustee – American College of Cardiology; and unfunded research – FlowCo. Samin K. Sharma has received honoraria as a speakers bureau member from Abbott Vascular, Boston Scientific, and Cardiovascular Systems Inc. The other authors reported no financial interests.
