# Supplementary material for: Sex-Specific Coronary Artery Calcium Score Threshold Predictive of Obstructive Coronary Artery Disease
Source: J Soc Cardiovasc Angiogr Interv. 2025 Dec 18;5(1):104048. doi: 10.1016/j.jscai.2025.104048 (PMC13033808; doi:10.1016/j.jscai.2025.104048)
Supplement: Supplementary Material [file mmc1.docx]

**Supplemental Figure 1.** Kappa test for quantitative coronary angiography versus obstructive CAD in clinical practice in our study


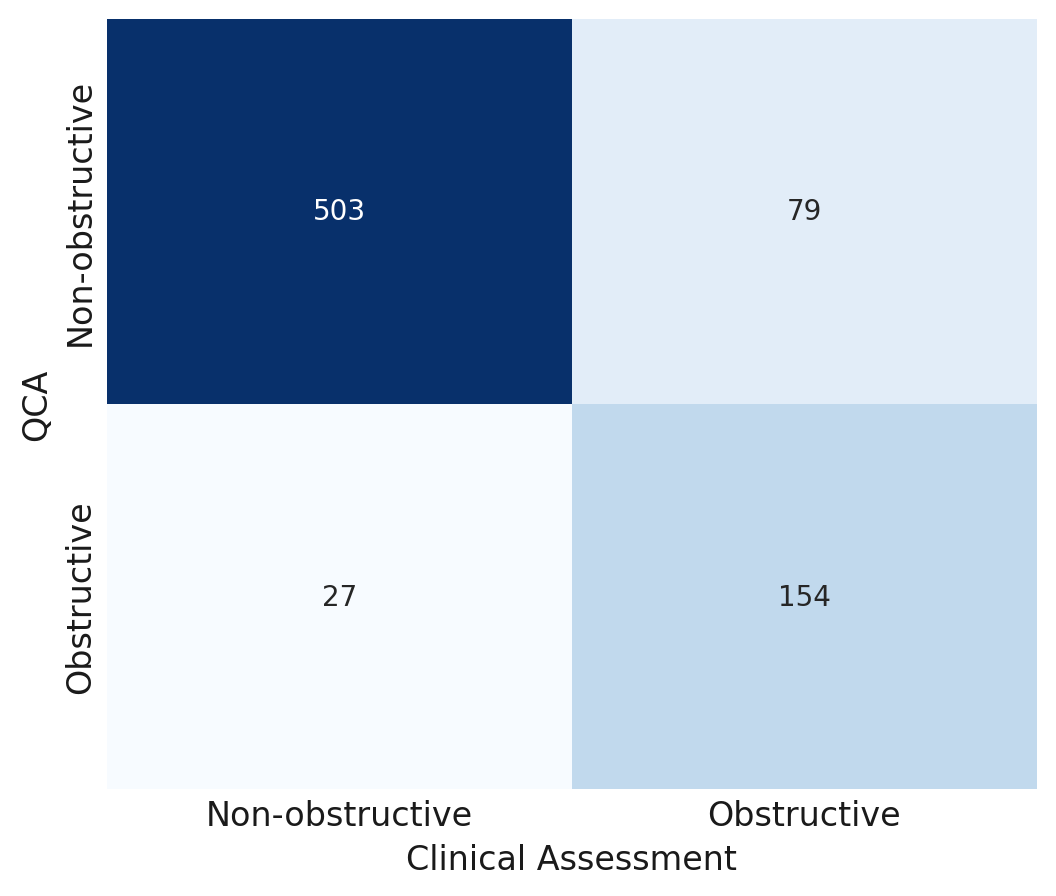


Abbreviations: QCA = quantitative coronary angiography

**Supplemental Figure 2.** Age sub-group analysis by CACS≥1400 for predicting obstructive CAD in men

**
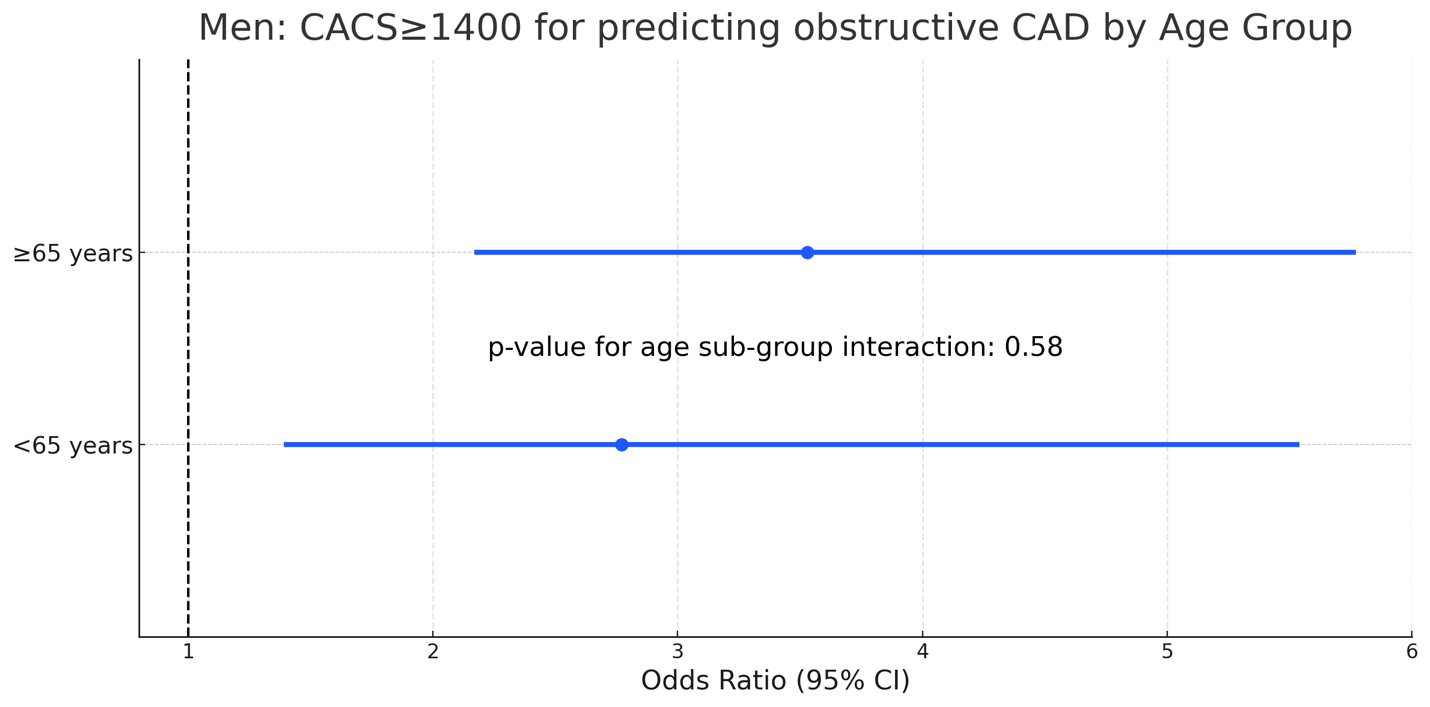
**

**Supplemental Figure 3.** Age sub-group analysis by CACS≥1000 for predicting obstructive CAD in women

**
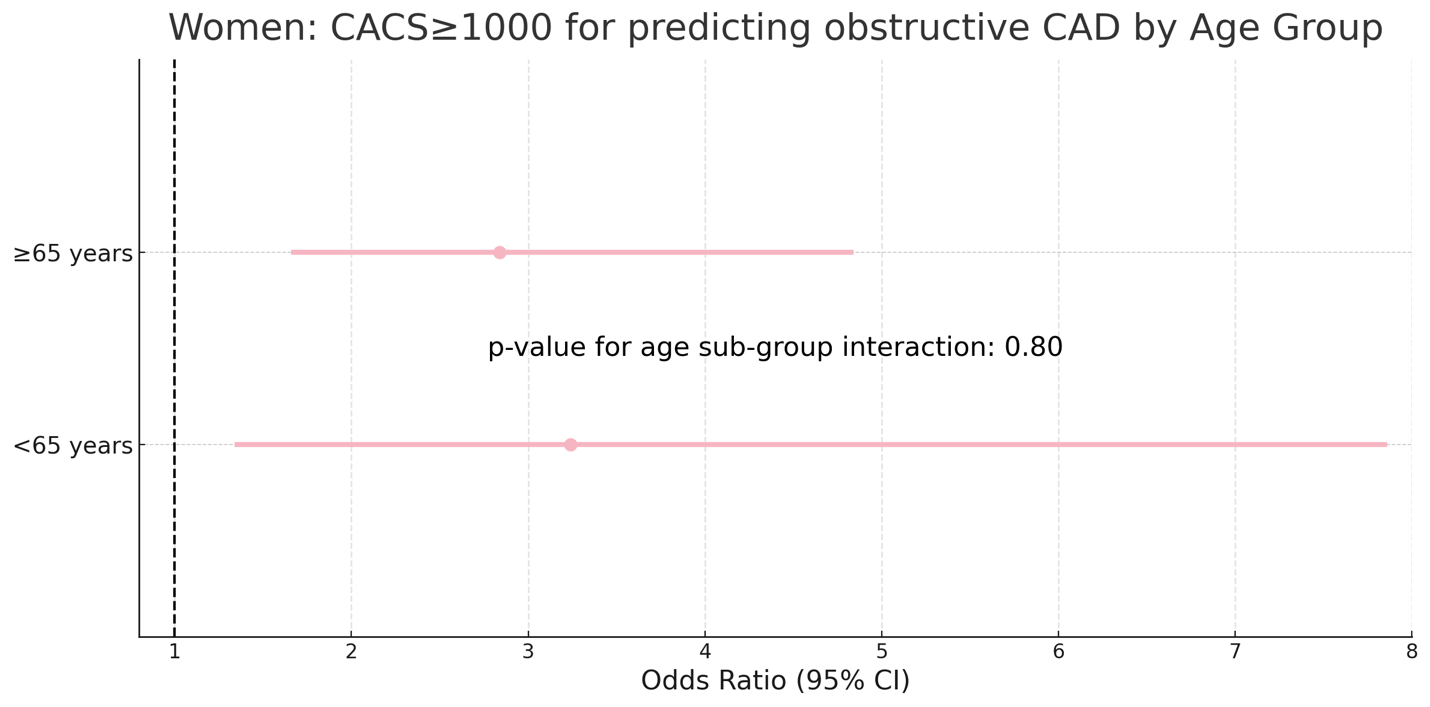
**

Abbreviations: CACS = coronary artery calcium score; CAD = coronary artery disease; CI = confidence interval
